# Should orthopedic surgeons consider the effects of gabapentin administration on bone healing while treating a long bone fracture: experimental study in a rat model

**DOI:** 10.1051/sicotj/2016028

**Published:** 2016-11-01

**Authors:** Hakan Sofu, Nizamettin Kockara, Bahattin Kerem Aydin, Bahadir Suleyman, Mahir Tayfur, Ismail Malkoc

**Affiliations:** 1 Faculty of Medicine, Erzincan University Erzincan 24030 Turkey; 2 Faculty of Medicine, Selcuk University Konya 42075 Turkey; 3 Faculty of Medicine, Atatürk University Erzincan 25030 Turkey

**Keywords:** Gabapentin, Fracture healing, Rat model

## Abstract

*Objective*: The main purpose of the present study was to assess the radiographic, histological, and mechanical effects of gabapentin on fracture healing in a rat model of femur fracture.

*Materials and methods*: A standard transverse fracture of the mid-diaphysis was created. A total of 60 female Wistar-Albino rats with the mean age of 13.5 ± 1.2 weeks were used for this experimental trial. The rats were randomized into four groups with 15 animals included in each group. Group A and B were the control groups whereas C and D were the treatment groups. Drugs were delivered by oral gavage twice a day with the daily dosage calculated according to body surface area conversion to the human equivalent dosing regimen of 1200 mg/day. Radiographic, histological, and biomechanical evaluation was performed.

*Results*: We could not detect any statistically significant difference between the control and gabapentin treatment groups according to the comparative assessment of radiographic scores on the 15th and 30th days. Although no significant differences were found between the groups on the 15th day, histological scores were better in the control group on the 30th day. According to the results of biomechanical testing, the fractured femurs resected from the control group exhibited significantly more strength on the 30th day.

*Conclusions*: According to the data we acquired during the present study, administration of gabapentin negatively affects the fracture healing process especially in the aspects of histological progression as well as the biomechanical strength of the callus in a rat model.

## Introduction

Bone healing is one of the unique repair processes of human body resulting in the union of fractures via the optimal reconstitution of the injured tissue to its original form without the formation of a scar [1–3]. It has not yet been completely elucidated and still remains as one of the most important topics in the field of orthopedics and traumatology [2, 4, 5]. A number of factors affect the healing which can be identified from both clinical and experimental work and may be taken into consideration to put treatment on a more rational basis [1]. As previously mentioned in the literature, improving knowledge about the factors that influence fracture healing not only helps to avoid the use of those that have a negative impact on the process but also enables the use of those that can induce a faster and more qualified union [6]. For that purpose, several experimental studies testing the positive or negative effects of different drugs or chemicals on fracture healing have been published in the literature [2, 6–12].

Gabapentin, 1-(aminomethyl)cyclohexaneacetic acid, is a structural analog of the neurotransmitter, γ-aminobutyric acid (GABA), which was introduced in 1993 as an adjuvant antiepileptic drug for the treatment of refractory partial seizure [13]. In today’s clinical practice, it is a widely used drug for the treatment of diabetic peripheral neuropathy, reflex sympathetic dystrophy, post-herpetic neuralgia, fibromyalgia, and neuropathic arthropathies. It has also been demonstrated that gabapentin had anti-nociceptive, anti-hyperalgesic, and anti-allodynic properties in clinical use [14]. Gabapentin may also be a useful option in order to reduce immediate post-operative pain and opioid consumption [15, 16]. Some studies have reported that it could be associated with a decrease in bone mineral density and could also increase nontraumatic fractures in those aged over 50 years [17, 18]. On the other hand, the effects of gabapentin on fracture healing process have not been evaluated yet in the literature.

The main purpose of the present study was to assess the radiographic, histological, and mechanical effects of gabapentin on bone healing in a rat model of femur fracture.

## Materials and methods

### Animals

This study has been reviewed and approved by the Ethical Research Review Board and the Experimental Animals Care and Use Committee. The study was conducted according to the Guide for the Care and Use of Laboratory Animals. A total of 60 female Wistar-Albino rats were used for this experimental trial. The mean age of the rats was 13.5 ± 1.2 weeks and their mean body weight was 233 ± 14 g. All animals were fed in the laboratory for a week before the operation in order to make them adapted to the new environment. Five rats were housed in each cage and provided fresh water and chow ad libitum with a 12–12 hour light-dark cycle.

### Fracture model and operative procedure

Following general anesthesia induced by the intraperitoneal administration of ketamine combined with xylazine, the rat was taken onto the operating table. A single dose antibiotic prophylaxis was administered (cefazolin sodium, 5 mg). The left thigh of the rat was prepared with povidone-iodine solution. One centimetre longitudinal skin incision was made over the lateral aspect of the left thigh and the soft tissues were dissected to expose the femoral shaft. A standard transverse fracture of the mid-diaphysis was created via multiple drilling followed by osteotomy. Then, the knee joint was incised and the patellar tendon displaced medially so as to expose the femoral condyles for the insertion of retrograde intramedullary Kirschner wire (0.8 mm diameter steel K-wire). The intramedullary K-wire was advanced up to the trochanter major using an electric drill, then it was slightly retracted, cut, and reinserted as both ends of the K-wire were located inside the bone. The incisions were sutured. No restriction of weight bearing or use of the operated limb was applied.

### Groups and drug treatment

The rats were randomized into four groups as A, B, C, and D with 15 rats included in each group. Group A and B were the control groups without any drug administration whereas C and D were the treatment groups. Drugs were delivered by oral gavage twice a day with the beginning from the end of the 4th postoperative hour. The daily dosage of gabapentin for each rat in Group C and D was calculated according to body surface area conversion to the human equivalent dosing regimen of 1200 mg/day. The rats in the control group (Group A and B) were also administered 1% methylcellulose (2 mL/day) by oral gavage twice a day at the same time periods as the rats in treatment groups in order to standardize the stress factor for all animals. The rats in Group A and C were sacrificed via cervical dislocation after anesthetic (ketamine) administration on the 15th postoperative day, and the rats in Group B and D on the 30th postoperative day. After the rats were killed, their left femur was disarticulated from the hip and knee joints. Soft tissues on the femoral bone were dissected off gently from the bone without any damage to the callus tissue. Radiographic evaluation was performed in all of the fractured femora. Histological examination was performed in half of the fractured femora from each group whereas the biomechanical evaluation was performed in the other half. None of the fractured femora was subjected to both mechanical and histological testing. The intact right femora were also harvested for comparative mechanical testing. During the intervention, animals in which there was evidence of fixation loss or infection were excluded from the study and humanely euthanized.

### Radiographic evaluation

Direct anteroposterior (AP) and lateral radiographic images of the sacrificed femurs were obtained on the 15th and 30th postoperative days. The radiographs were evaluated and the union status was classified according to the Lane and Sandhu grading system [19]. Radiographic scoring was achieved by two of the authors and the lowest correlation coefficient was 0.88.

### Histological evaluation

The soft tissues covering the fractured femora were dissected without removing the periosteum and the K-wire was carefully removed without any damage to the callus tissue. The femurs were fixed in 4% paraformaldehyde in phosphate-buffered saline solution at 4 °C for two days before undergoing decalcification in 7% formic acid. Following decalcification process, specimens were then embedded in paraffin block and 7 μm sections were cut. Hematoxylin and eosin staining was applied. Slides were assessed under light microscope. The callus tissue was scored based on the system recommended by Huo et al. [20].

### Mechanical testing

Following the dissection and disarticulation, the femora were immediately wrapped in saline-soaked gauze, double-bagged, and placed in a −20 °C freezer. The night before mechanical testing, the samples were thawed overnight in an 8 °C refrigerator. In order to determine the biomechanical behaviour of test groups, three point bending tests were performed. The specimens were placed on the three point bending test apparatus in the lateral direction. The distance between the rollers was selected as 30 mm considering specimen sizes. A preload of 5N for all specimens was applied before tests and then the specimens were subjected to axial compressive forces until fracture occurred. The biomechanical tests were performed using a Shimadzu testing machine with a loading rate of 2 mm/min at room temperature. Mechanical tests were performed both for the intact right femur and the fractured left femur from the control and treatment groups.

### Statistical analysis

Statistical analysis was performed with the Kruskal-Wallis test in intergroup comparisons, the Mann-Whitney U-test in pair group comparisons, and Fisher’s exact test in the comparison of qualitative data. Significance in the results was evaluated at the level of *p* < 0.05.

## Results

We did not observe any complications related to the anesthetic or oral drug administration. Two rats in Group A, one rat in Group C, and one rat in Group D were humanely euthanized due to infection. Loss of fixation was diagnosed in two rats from Group B and one rat from Group D, and they were also euthanized. The study was started with 15 rats included in each of the four groups and data analysis included 13 rats in three of the groups (A, B, D) and 14 rats in one group (C). Seven fractured femora underwent histological evaluation in each group, whereas six femora from Group A, B, and D with seven femora from Group C underwent biomechanical testing.

**Figure 1.**
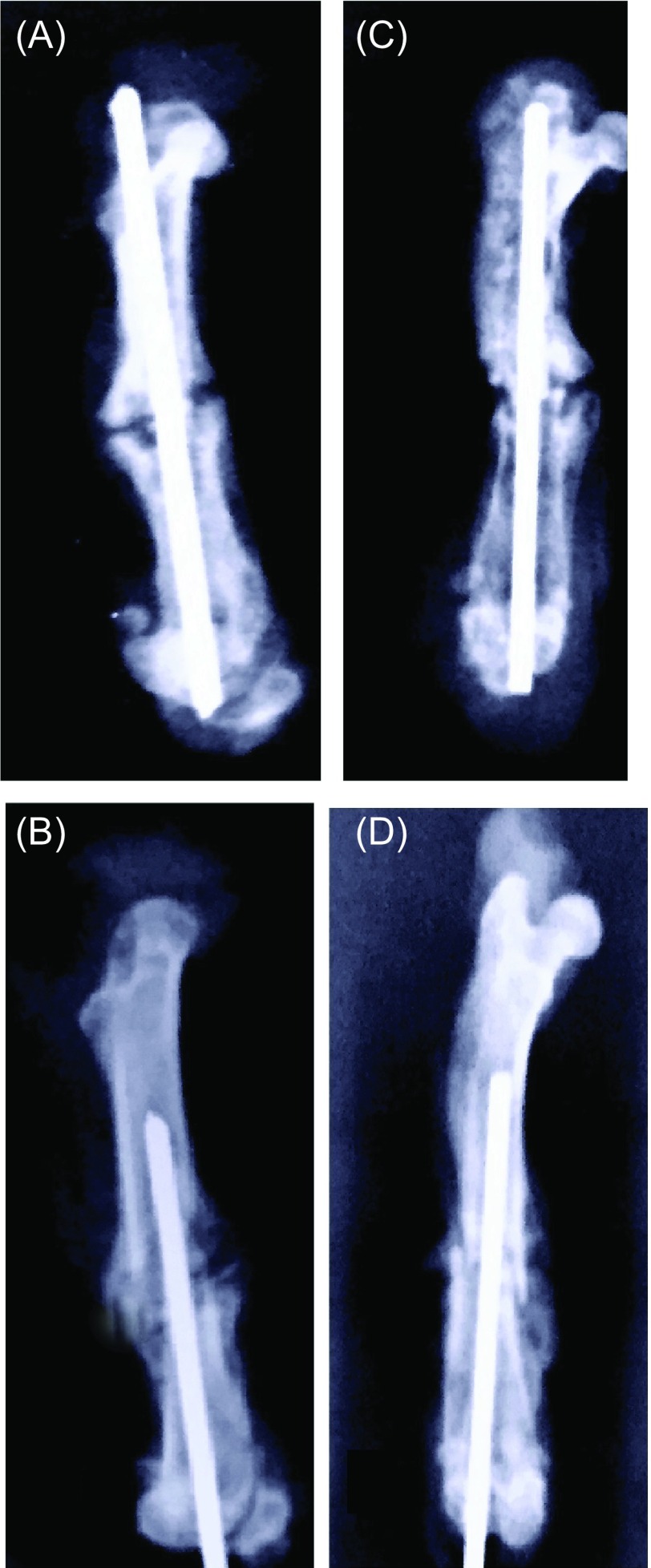
Radiographic progression of fracture healing. **A**: Radiographic image of a fractured femur from Group A on the 15th day. **B**: Radiographic image of a fractured femur from Group B on the 30th day. **C**: Radiographic image of a fractured femur from Group C on the 15th day. **D**: Radiographic image of a fractured femur from Group D on the 30th day.

Significant radiographic improvements were noted in both the control and gabapentin treatment groups from the 15th to 30th day after fracture (Figure 1). However, we could not detect any statistically significant difference between the control and gabapentin treatment groups according to the comparative assessment of radiographic scores on the 15th and 30th days (*p* = 0.279 and *p* = 0.075, respectively) (Table 1).

**Table 1.** Comparison of radiographic scores between the control and treatment groups.

Group	Mean (*SD*)		*p* value
	Day 15	Day 30	
Control	3 ± 0.69	4.25 ± 0.75	0.003
Gabapentin	2.66 ± 0.53	3.5 ± 0.81	0.028
*p* value	0.279	0.075	

**Figure 2.**
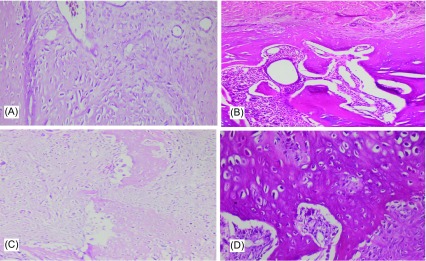
Histological progression of fracture healing. **A**: Predominant cartilage tissue with less fibrous tissue (Grade 3) in a fractured femur from Group A on the 15th day. **B**: Immature bone tissue with cartilage tissue in a uniform manner (Grade 7) in a fractured femur from Group B on the 30th day. **C**: Wide area of fibrous tissue with less cartilage tissue (Grade 2) in a fractured femur from Group C on the 15th day. **D**: Wide area of cartilage tissue (Grade 5) in a fractured femur from Group D on the 30th day.

According to the histological evaluation performed under a light microscope using hematoxylin and eosin staining, we did not observe any foreign body reactions, findings of histotoxicity or inflammation, and cortical bone necroses in neither the control group nor the gabapentin treatment group. During the examinations, the presence of fibrous tissue, cartilage tissue, or immature bone was assessed. Significant histological improvements were noted in both the control and gabapentin treatment groups according to the evaluation of the specimens on the 15th and 30th days (Figure 2). However, although no statistically significant differences were detected between the groups on the 15th day (*p* = 0.063), histological scores were better in the control group in comparison to the gabapentin treatment group on the 30th day after fracture (*p* = 0.005) (Table 2).

**Table 2.** Comparison of histological scores between the control and treatment groups.

Group	Mean (*SD*)		*p* value
	Day 15	Day 30	
Control	3.87 ± 0.81	7.37 ± 0.53	<0.001
Gabapentin	3.11 ± 0.69	6 ± 0.98	<0.001
*p* value	0.063	0.005	

**Figure 3.**
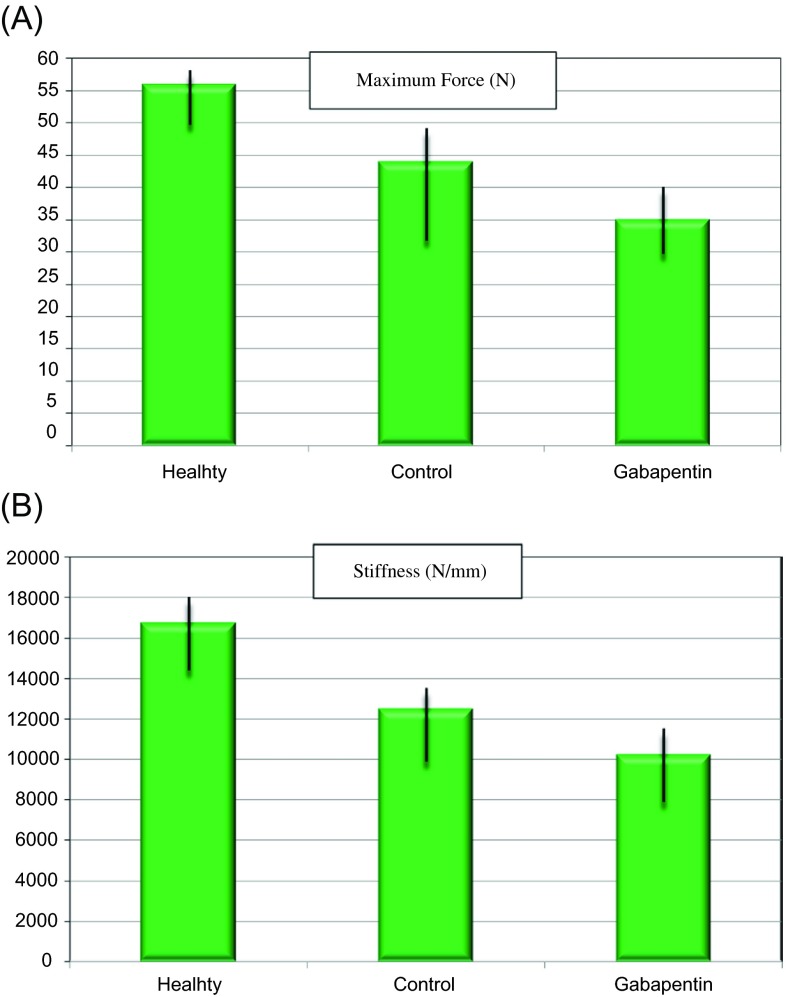
(A and B): The maximum force and stiffness values of the intact femora and fractured femora from control and gabapentin treatment groups on the 30th day.

The data acquired via biomechanical testing demonstrated that the intact right femora exhibited significantly more strength than all of the fractured left femora (*p* < 0.001). There was no difference between the strength of the non-fractured right femora from control and gabapentin treatment groups. On the other hand, statistically significant differences of maximum force (*p* = 0.030) and stiffness (*p* = 0.002) between the control and gabapentin treatment groups were detected. According to the results of biomechanical testing, the fractured femora resected from the control group exhibited significantly more strength than the femora resected from the gabapentin treatment group on the 30th day (Figures 3A and 3B).

## Discussion

The fracture healing process is composed of complex biological events which progressed via the specific activities of hematopoietic and immune cells as well as mesenchymal stem cells recruited from the surrounding tissues and the circulation [2, 4]. It has been demonstrated that many different local and systemic factors influence this special repair mechanism of the human body [21, 22]. Experimental studies continue to be one of the most important tools to test these factors and provide basic information to developing clinical practice. Several studies that mainly focused on and discussed the effects of various drugs on bone healing process have been published in the literature [2, 5–12, 23]. Accelerating the union of a fractured bone has been the major purpose of such studies as well as identifying the agents that negatively affect its progression [5–7, 23]. Although the effects of gabapentin on wound healing in rats [24] and intestinal incision wound healing in rabbits [25] have been tested, no study has examined the direct relationship between bone metabolism and gabapentin monotherapy. Therefore, the main purpose of the present study was to assess the radiographic, histological, and mechanical effects of gabapentin on bone healing in a rat model of femur fracture.

Gabapentin might be used for a wide range of clinical indications and may also be administered to control acute and chronic pain syndromes as well as to reduce postoperative pain and opioid consumption after various surgical procedures [26]. Gabapentin reduces pain transmission and central sensitization via binding to the alpha-2-delta subunit of presynaptic voltage-gated calcium channels, inhibiting calcium influx and attenuating glutamate release in the nociceptive pathways [15]. Either as an ongoing medical treatment of accompanying neurological disorders or as an effective agent administered to control pain, many patients treated for a fracture during daily clinical practice of traumatology have been using gabapentin. Jette et al. reported that most of the antiepileptic drugs including gabapentin were associated with an increased risk of nontraumatic fractures in individuals aged 50 years or older [18]. Verrotti et al. also mentioned that long-term gabapentin consumption leads to a decrease in bone mineral density [17]. Besides, a prospective study conducted by Ensrud et al. confirmed that it could induce bone loss at the femoral neck [27]. On the other hand, as emphasized by Meier and Kraenzlin, although gabapentin has been demonstrated to induce bone loss it has remained unclear whether gabapentin has direct deleterious effect on bone or whether the increased fracture risk might be attributed to decreased mobility as it is frequently used to treat chronic pain syndromes [28]. As the most important finding of our study, although radiographically no significant difference was detected between the control and gabapentin treatment groups on the 15th and 30th days after fracture, gabapentin was found to have negative effects on the histological progression of bone healing as well as on the biomechanical strength of the callus tissue. Unfortunately, because we did not perform any molecular evaluation based on the cytokines, signaling pathways, or bone markers we were unable to explain what kind of mechanism could have resulted in those negative effects.

The major limitation of the present study was the limited number of animals included in each group. As a second limitation, we did not make any comparison based on the serum levels of cytokines and bone markers. Thirdly, radiographic assessment could not be performed using a micro-CT device which was not available in our institute. On the other hand, the effects of gabapentin were assessed via radiographic, histological, and mechanical analysis of the fracture healing process. The standardized experimental fracture healing model applied in this study has been widely used in the literature [5–7, 29]. Standard drug administration was achieved by oral gavage twice a day by the same administrator at the same time every day of the study period. The dosage was calculated according to body surface area conversion which was recommended as the standard way to approximate equivalent exposure of drugs among different kind of animals [30]. Additionally, administration of 1% methylcellulose (2 mL/day) by oral gavage in the control group standardized the stress factor for all animals. We applied biomechanical testing of the intact right femora, fractured left femora without any drug administration, and fractured left femora with oral gabapentin treatment to achieve the best objective comparison in the aspect of mechanical strength.

In conclusion, although the results of experimental animal studies cannot be directly adapted to daily clinical practice; the data we acquired during the present study demonstrated that administration of gabapentin negatively affects fracture healing process especially in the aspects of histological progression as well as the biomechanical strength of the callus in a rat model. This study may be a reference for further experimental and clinical trials which are required to establish a better understanding of the clinical relevance of our findings.

## Conflict of interest

There were no conflicts of interest regarding the submission and publication of this manuscript.
